# Sperm Chromatin Dispersion Test Detects Sperm DNA Fragmentation Mainly Associated with Unviable Spermatozoa and Underestimates the Values with Respect to TUNEL Assay

**DOI:** 10.3390/ijms25084481

**Published:** 2024-04-19

**Authors:** Maria Emanuela Ragosta, Giulia Traini, Lara Tamburrino, Selene Degl’Innocenti, Maria Grazia Fino, Sara Dabizzi, Linda Vignozzi, Elisabetta Baldi, Sara Marchiani

**Affiliations:** 1 Department of Experimental and Clinical Medicine, University of Florence, 50139 Florence, Italy; mariaemanuela.ragosta@unifi.it; 2 Department of Experimental and Clinical Biomedical Sciences “Mario Serio”, University of Florence, 50139 Florence, Italy; giulia.traini@unifi.it (G.T.); linda.vignozzi@unifi.it (L.V.); sara.marchiani@unifi.it (S.M.); 3 Andrology, Women’s Endocrinology and Gender Incongruence Unit, Center for Prevention, Diagnosis and Treatment of Infertility, Careggi University Hospital, 50134 Florence, Italy; lara.tamburrino@unifi.it (L.T.); deglinnocentis@aou-careggi.toscana.it (S.D.); finomg@aou-careggi.toscana.it (M.G.F.); dabizzis@aou-careggi.toscana.it (S.D.)

**Keywords:** spermatozoa, sperm DNA fragmentation, SCD (sperm chromatin dispersion) test, TUNEL, TUNEL/PI

## Abstract

Several clinical laboratories assess sperm DNA fragmentation (sDF) in addition to semen analysis in male infertility diagnosis. Among tests evaluating sDF, TUNEL (Terminal deoxynucleotidyl transferase dUTP nick end labeling) and SCD (Sperm Chromatin Dispersion) are widely used. Our lab developed a modified version of TUNEL (TUNEL/PI) able to distinguish two sperm populations (PI Brighter and PI Dimmer) differently associated with sperm viability and reproductive outcomes. The aim of this study was to compare sDF levels detected by SCD and TUNEL/PI in the semen samples from 71 male subjects attending our Andrology Laboratory. Our results demonstrate that SCD is less sensitive in determining sDF compared to TUNEL/PI. The statistically significant positive correlation found between sDF evaluated by SCD and PI Dimmer (consisting of all dead spermatozoa) suggests that SCD mainly detects sDF in unviable spermatozoa. We confirmed that most spermatozoa detected by SCD are unviable by performing SCD after incubation in hypo-osmotic medium to discriminate viable and unviable cells in 52 samples. Such results might explain the lower ability of this test in discriminating couples having successful ART outcomes demonstrated in published metanalyses. Overall, our results indicate that SCD is less sensitive in evaluating sDF for diagnostic purposes.

## 1. Introduction

Sperm DNA fragmentation (sDF) is a common alteration of paternal genetic material consisting of single- and double-DNA strand breaks, mainly occurring in subfertile subjects [1] and attracting attention as a potential cause of paternal anomalies transmitted to offspring [2]. Several exogenous factors could provoke sDF, including uncorrected lifestyle habits, advanced paternal age, pathologies, genital tract infections, exposure to environmental toxicants, and exposure to chemo- and radiotherapies [3]. Protamination failure, abortive apoptosis and oxidative stress represent the main endogenous mechanisms responsible for DNA damage [4,5,6].

Several studies have investigated the association between DNA integrity and reproductive outcomes, finding that high levels of sDF reduce the chances of achieving pregnancy via natural conception [7], after intrauterine insemination (IUI) [8,9] as well as in vitro fertilization (IVF) and intracytoplasmic sperm injection (ICSI) [10,11,12]. Moreover, sperm DNA damage is associated with increased miscarriage rates [10,13] in IVF/ICSI cycles as well as with recurrent pregnancy loss in both natural [14,15] and assisted reproduction [16,17].

sDF is presently evaluated in several clinical laboratories to add information on sperm molecular aspects not revealed by routine semen analysis. Indeed, although the latter is still considered the cornerstone for male infertility diagnosis [18], it does not discriminate fertile from infertile subjects since seminal parameters overlap between these categories [19]. Accordingly, the last edition of the WHO laboratory manual for the examination and processing of human semen [20] included sDF among the extended semen evaluations to be performed for diagnostic purposes in certain clinical circumstances, describing the most popular methods that can be used to evaluate it. In particular, four methods are mentioned [20], namely, two assays that directly evaluate the presence of DNA breaks, i.e., TUNEL (terminal deoxynucleotidyl transferase-mediated fluorescein-dUTP nick end labelling) and COMET (or SCGE, single-cell gel electrophoresis), and two assays that evaluate chromatin susceptibility to denaturation, i.e., SCSA (sperm chromatin structure assay) and SCD (sperm chromatin dispersion) [21]. None of them are standardized and there are no validated cut-off values [20,22]. In addition, although the results of the four tests appear to correlate [23], they assess different aspects of sperm DNA integrity.

TUNEL and SCD assays are widely used for clinical purposes. By using the TUNEL assay, DNA fragmentation is revealed by labelling the 3′ free ends of DNA with fluorescent dUTP by the terminal deoxynucleotidyl transferase enzyme. Fluorescence can be detected both by flow cytometry and microscopy. The TUNEL method coupled with flow cytometry allows a rapid analysis of a large number of cells in a short period of time and provides a highly sensitive, precise and objective tool to define spermatozoa with fragmented DNA. A few years ago, our laboratory developed a modified version of TUNEL by including the addition of the nuclear stain Propidium Iodide (PI) [24]. This method, named TUNEL/PI, allows for discriminating spermatozoa from semen interferents as well as distinguishing two sperm populations (PI Brighter and PI Dimmer) that are differently associated with sperm viability (PI dimmer is composed of 100% unviable and DNA-fragmented spermatozoa, whereas PI Brighter contains both viable and unviable DNA-fragmented spermatozoa [25]) and other seminal parameters [24]. In addition, only the PI Brighter population was able to distinguish between fertile and sub-fertile men [26], indicating that DNA damage in this population better associates with reproductive outcomes. SCD is based on the principle that, after acid denaturation and the removal of nuclear proteins, spermatozoa with intact DNA produce the characteristic “halo” of dispersed DNA loops, which is not observed in spermatozoa with damaged DNA. The procedure is easy to perform and spermatozoa with or without haloes can be observed by optical or fluorescent microscopy. Clearly, when evaluation is made by microscopy, results are operator-dependent and a smaller number of spermatozoa are analyzed with respect to flow cytometric analysis. Therefore, the results might be less accurate, reproducible and objective. On the other side, evaluation by flow cytometry requires sophisticated and expensive instrumentation. These characteristics induce many laboratories performing assisted reproductive technology (ART) to prefer the use of SCD for routine analysis. However, according to published meta-analyses [11,27,28], SCD appears less valuable than TUNEL in revealing the sperm DNA damage that impacts ART outcomes, indicating that the damage revealed by SCD may be less relevant for reproductive purposes or that the sperm population analyzed is less involved. Taking advantage from the use of the TUNEL/PI method that distinguishes two sperm populations with different reproductive impact [24,26], we evaluated sDF levels in the same semen specimens by using SCD and TUNEL/PI to compare the results obtained with the two methods.

## 2. Results

**Figure 1:**
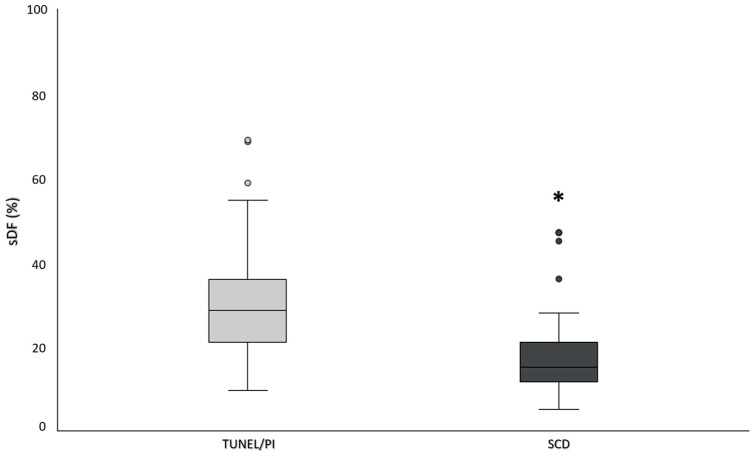
Box plots representing the percentage of sDF evaluated by TUNEL/PI method and SCD test in total sperm population (N = 71). *: significance, *p* < 0.001 vs. total sDF (TUNEL/PI). Dots represent outlier values.

After performing both TUNEL/PI and SCD in the same 71 semen samples, a statistically significant difference in the percentage of spermatozoa with fragmented DNA was observed between the two methods (Figure 1). In particular, the percentage of sDF in the total sperm population was significantly higher when detected by TUNEL/PI with respect to that determined by SCD (median values [IQR]: 28.6% [20.6–36.9] for TUNEL/PI vs. 15.0% [11.0–21.0] for SCD, *p* < 0.001, N = 71).

**Figure 2:**
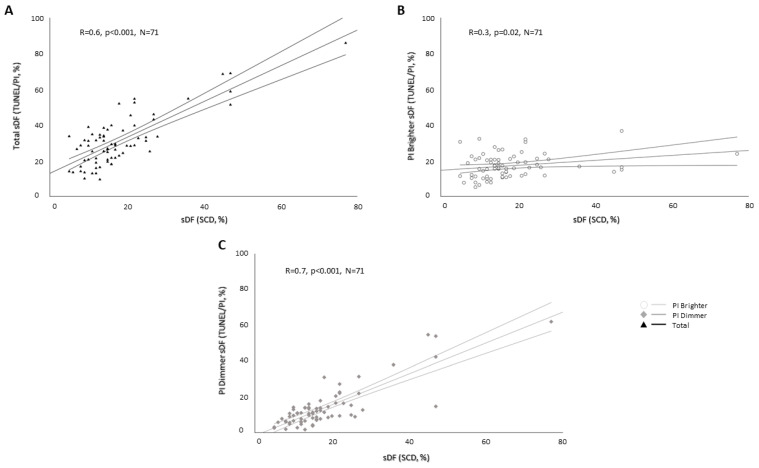
Correlations between the percentage of DNA-fragmented spermatozoa evaluated by SCD test and total (**A**), PI Brighter (**B**) and PI Dimmer (**C**) sDF evaluated by TUNEL/PI method. R: Spearman’s correlation coefficient; *p*: significance; N: number of subjects.

The sDF levels evaluated by SCD positively correlated with those obtained by TUNEL/PI in the total sperm population (R = 0.6, *p* < 0.001, N = 71, Figure 2A). When distinguishing PI Brighter and PI Dimmer sperm populations, a strictly positive correlation was observed between the percentage of DNA-fragmented spermatozoa revealed by SCD and that of PI Dimmer (R = 0.7, *p* < 0.001, N = 71, Figure 2B), whereas a weak association was found with PI Brighter (R = 0.3, *p* = 0.02, N = 71, Figure 2C).

**Figure 3:**
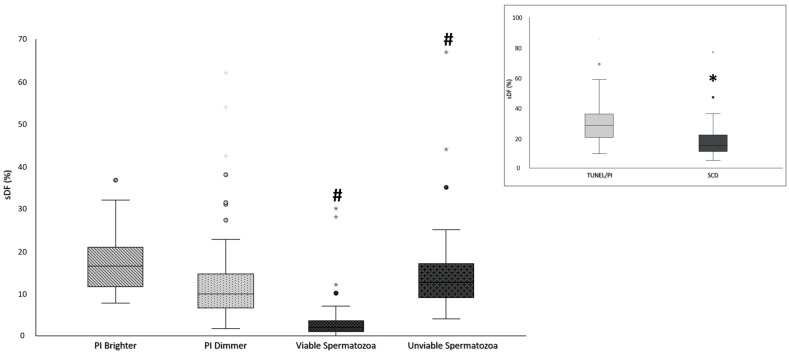
Box plots representing sDF in PI Brighter and PI Dimmer populations (evaluated by TUNEL/PI method) as well as in viable and unviable populations (evaluated by HOS/SCD test). In the insert, box plots representing sDF were evaluated by TUNEL/PI method and SCD test for total sperm population (N = 52). *: significance, *p* < 0.001 vs. Total sDF; # *p* < 0.01 vs. PI Brighter sDF. Dots represent outlier values.

Since, as mentioned above, PI Dimmer population is composed by 100% unviable spermatozoa [25], the high correlation of sDF values detected with SCD and PI Dimmer suggests that SCD tests may reveal sDF mainly in unviable spermatozoa. To investigate this hypothesis, we modified the protocol of SCD, by incubating spermatozoa in hypo-osmotic swelling medium before performing the SCD procedure (HOS/SCD test) in order to discriminate viable (spermatozoa showing curled tails) and unviable (spermatozoa without curled tails) cells allowing the identification of four sperm patterns (as described in Materials and Methods). sDF detected by both TUNEL/PI and modified HOS/SCD were compared in 52 semen samples. The results confirmed that sDF values by the HOS/SCD test were lower with respect to the TUNEL/PI method in the total sperm population (median values [IQR]: 28.4% [20.3–36.4] for TUNEL/PI vs. 15.0% [11.0–22.0] for HOS/SCD, *p* < 0.001, N = 52, Figure 3, insert). After dividing in viable and unviable populations, most spermatozoa showing DNA damage by HOS/SCD resulted to be unviable (median values [IQR]: 12.5% [9.0–17.0] for unviable vs. 2.0% [1.0–3.8] for viable, *p* < 0.001, N = 52, Figure 3) and the percentage of unviable cells with fragmented DNA was not significantly different from that observed in the PI Dimmer population with TUNEL/PI (Figure 3).

**Figure 4:**
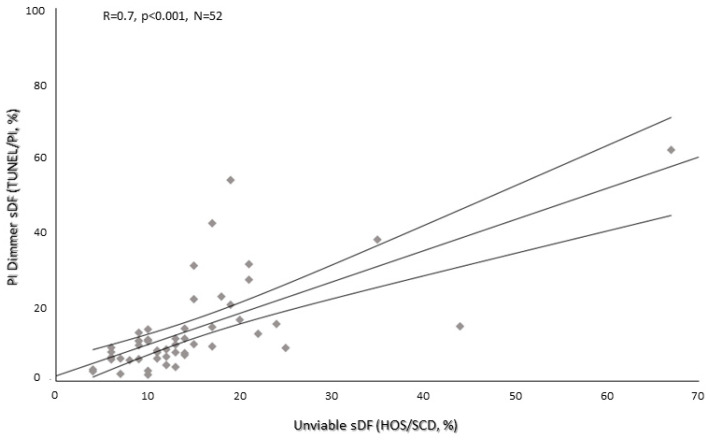
Correlation between the percentage of unviable DNA-fragmented spermatozoa (evaluated by HOS/SCD test) and PI Dimmer sDF (evaluated by TUNEL/PI method). R: Spearman’s correlation coefficient; *p*: significance; N: number of subjects.

PI Dimmer and unviable HOS/SCD sDF values were correlated (r = 0.7, *p* < 0.001, N = 52, Figure 4). Moreover, the percentage of unviable cells with fragmented DNA was significantly lower with respect to PI Brighter sDF (median values [IQR]: 12.5% [9.0–17.0] for unviable HOS/SCD vs. 16.4% [11.5–21.0] for PI Brighter, *p* < 0.01, N = 52, Figure 3).

## 3. Discussion

It is now an established concept that infertility is a couple’s problem and does not just concern one of the two partners. Indeed, attention has also begun to be paid to the fertility status of the male partner, often overlooked in the past. However, the standard semen analysis is a poorly predictor of the success in reproductive outcomes; thus, other tests providing additional information on sperm quality and functions can be employed by clinical laboratories. The evaluation of sperm DNA integrity can be of particular interest since compelling evidence indicates that sDF can affect natural and assisted pregnancy achievement, impairing embryo development and, ultimately, leading to miscarriage [29]. Four main methods are currently used to detect sDF, however, none of these is currently indicated as “gold standard”. They reveal different types of DNA damage, are not standardized and each laboratory should establish its own reference values based on the method and protocol used [20,22]. All these factors have limited the broad application of these tests in routine practice, although sDF is evaluated in many clinical laboratories to improve male infertility diagnosis. Here, we demonstrated that two popular assays which evaluate sDF, TUNEL and SCD cannot be used indiscriminately. In particular, we show, comparing the percentage of DNA-fragmented spermatozoa revealed by SCD with that by TUNEL/PI, that SCD detects only a part (on average about 54%) of sDF found by our method. Importantly, we show here that most sDF by SCD is detected in unviable cells, as indicated by the strong association with PI Dimmer population (that is, the population made up of spermatozoa that are all dead and DNA-fragmented, [24,25]) and by experiments where SCD was associated with the HOS test, allowing us to distinguish sDF in viable and unviable spermatozoa. Overall, these findings might explain the lower association with reproductive outcomes derived from meta-analyses of SCD data [11,27,28]. Indeed, unviable spermatozoa have low/no probability in participating in successful reproductive outcomes. Consistently, we demonstrated previously that the sDF-PI Dimmer population does not discriminate between fertile and sub-fertile subjects compared to PI Brighter [26]. As mentioned above, SCD provides an indirect measure of sDF by evaluating susceptibility to acidic denaturation [30]. We cannot exclude the fact that viable spermatozoa, even if with fragmented DNA, are less susceptible to acidic denaturation.

Previous studies comparing SCD and TUNEL reported a good correlation between them and that both methods are similarly capable of distinguishing fertile and infertile men [21,31,32,33,34,35,36]. Our study confirms the occurrence of good correlations, at least, between SCD and total and PI Dimmer sDF evaluated by TUNEL/PI. Table 1 reports the results of studies comparing percentage sDF values evaluated with SCD and TUNEL in the same semen samples. Most studies [21,31,32,35] reported similar values of sDF with the two techniques; one study reported higher values for SCD [36], and the present and Grèze et al. [33] studies found lower SCD values with respect to TUNEL (Table 1). Of note, the methodological conditions were different in these studies. In particular, in the studies of Zhang et al. [31], Chohan et al. 2006 [34], Javed et al. [32] and Feijó and Esteves [36], the comparison was performed between SCD and TUNEL revealed by fluorescence microscopy. Conflicting results were previously reported in studies investigating TUNEL revealed, by flow cytometry or fluorescence microscopy; although a correlation between results obtained by the two techniques was found, the percentages of sDF levels were different [24,37,38]. In general, it is well known that microscopic determination of spermatozoa, whether processed by TUNEL, SCD or otherwise, is influenced by factors such as the subjectivity of the operator, the lower detection sensitivity of the eye with respect to that of the flow cytometry, and the small number of observations. In addition, it should be noted that Zhang et al. [31] performed simultaneous evaluation on the same slide of SCD and TUNEL, by first performing the SCD procedure and then labelling with TUNEL reagents. In this way, TUNEL was performed on acid-denatured samples. The study of Feijó and Esteves [36] is the only one, among those indicated in Table 1, which omits the permeabilization step in the TUNEL process, which is fundamental in allowing the entry of the staining solution in the cells, likely explaining why lower values were obtained with this method compared to SCD. In addition, in the studies by Ribas-Manou et al. [21] and Garcia-Peiro et al. [35], where the comparison was performed with respect to TUNEL coupled with flow cytometry as in our study, cytofluorimetric analysis was not performed by excluding semen interferents, in particular apoptotic bodies. The setting of an FSC/SSC region excluding debris and large cells and the use of TUNEL/PI double staining allow us to distinguish semen interferents from spermatozoa and to obtain a more accurate measure of sDF [24]. In particular, if apoptotic bodies are not omitted from the analysis, sDF levels are lower [24]. In addition, it should be pointed out that even small variations in the TUNEL method may result in important and significant differences in the percentages of sDF [39]. Finally, Ribas-Manou et al. [21] and Garcia-Peiro et al. [35] evaluated sDF in cryopreserved semen samples with both methods [21,35]. It has been reported in several studies that cryopreservation procedures can increase sDF levels and that different susceptibilities to damage are found in different semen samples (for rev see [40]).

Similar to the present study, Grèze et al. [33] found that SDF values detected by SCD tended to be significantly lower compared with those revealed by TUNEL in samples selected by density gradient centrifugation, and therefore their study was conducted in a different condition with respect to our study. The difference between the two methods, in sDF levels and in the total sperm population, was more evident in our study (−13% vs. −3.4% in the study by Grèze et al. [33]). Interestingly, our study also evidenced that the difference between PI Dimmer and sDF in unviable sperm by HOS/SCD was less than 1%, reinforcing the concept that SCD detects damage mostly in dead cells. On average, only 2% viable spermatozoa showed DNA fragmentation when detected with HOS/SCD, which is at variance with other studies detecting sDF in viable cells and reporting levels up to 20% [41,42].

## 4. Materials and Methods

### 4.1. Semen Samples Collection and Preparation

Semen samples were collected from 101 male subjects, with a minimum of two and a maximum of seven days of sexual abstinence, undergoing routine semen analysis for couple infertility at the Andrology Laboratory of Careggi University Hospital of Florence. The only inclusion criterion was the obtainment of signed informed consent to use the remaining semen after completion of the analysis. Semen analysis was carried out according to the WHO manual (VI edition, [20]). After 30 min from semen collection, volume, viscosity and pH were evaluated together with sperm concentration, motility, viability and morphology. Only semen samples with a sperm number ≥ 15 × 10^6^ (N = 71) were chosen for subsequent analyses by the TUNEL/PI assay and SCD test. The median values of age, days of sexual abstinence and basal semen characteristics of the 71 male subjects included in this study are reported in Table 2.

### 4.2. TUNEL/PI and Flow Cytometry

10 × 10^6^ spermatozoa were washed twice with Human Tubal Fluid (HTF, from Fujifilm Italia S.p.A., Milan, Italy) medium and fixed with paraformaldehyde (500 µL, 4% in phosphate-buffered saline solution [PBS], pH 7.4) for 30 min at room temperature. Then, the spermatozoa were washed twice with 200 µL of PBS with 1% BSA and permeabilized with 0.1% Triton X-100 in 100 µL 0.1% sodium citrate for 4 min in ice. After dividing each sample into two aliquots (test sample and negative control), the labelling reaction was performed by using In Situ Cell Death Detection Kit (Roche Molecular Biochemicals, Milan, Italy). The spermatozoa were incubated for 1 h at 37 °C in the dark in 50 µL of labelling solution (supplied by the kit) containing the TdT enzyme (1:10) for the test sample and only with the labelling solution for the negative control. Then, the samples were washed twice, resuspended in 300 µL of PBS, stained with PI (30 µg/mL) and incubated in the dark for 5 min at room temperature.

Samples were acquired by FACScan flow cytometer (Becton Dickinson Biosciences, San Jose, CA, USA) equipped with a 15 mW argon-ion laser for excitation. For each test sample, three sperm suspensions were prepared for instrumental setting and data analysis (1) by omitting both PI staining and TdT, (2) by omitting only TdT (negative control), and (3) by omitting only PI staining (for fluorescence compensation). Green fluorescence of TUNEL and red fluorescence of PI were revealed by FL-1 (515–555 nm wavelength band, voltage set 590) and FL-2 (563–607 nm wavelength band, voltage set 477) detectors, respectively. For each sample, 8000 events were recorded within the flame-shaped region characteristic of spermatozoa in the forward-light scatter/side-light scatter dot plot, and sDF was determined by gating the nucleated events (i.e., the events labelled with PI) within this region [24]. A marker, including 99% of total events, was set in the dot plot related to negative control and translated in the corresponding dot plot of the test sample, and all the events beyond the marker were considered positive for TUNEL. sDF was calculated both in PI Brighter and PI Dimmer populations and then added to obtain the percentage of total DNA-fragmented spermatozoa, as previously described [24].

### 4.3. SCD Test

SCD was performed by SpermFunc^®^DNAf kit (BRED Life Science, Shenzhen, China). Briefly, semen samples were diluted in HTF medium at a concentration of 3 × 10^6^/mL, and 60 µL were dispensed into the tube of dissolved agarose gel. Subsequently, 30 µL of sperm suspension was placed on the well of a pre-coated slide and leaving it at 2–8 °C for 5 min. The pre-coated slide was first incubated with the acid solution provided by the kit (solution A) at room temperature for 7 min and then in lysis solution (solution B) for 25 min at room temperature. After washing in distilled water for 5 min, the pre-coated slide was moved to 70% ethanol for two minutes, then to 90% ethanol for two minutes, and finally to 100% ethanol for two minutes. Staining was performed by dispensing 15–20 drops of Wright’s stain on the pre-coated slide and incubating for 15 min and then washing with water. Once the slide was air dried, a total of 200 spermatozoa were evaluated on each slide, with a 40× bright-field microscope (Nikon Eclipse Ci, Nikon Europe B.V., Amstelveen, The Netherlands). The score was performed according to the instructions provided by the manufacturer: spermatozoa with large or medium-sized halos were identified as non-fragmented cells (SCD+), whereas cells with small or no halos were identified as fragmented cells (SCD−) [43]. The percentage of DNA-fragmented spermatozoa was calculated dividing the number of SCD-spermatozoa on the total number of spermatozoa observed ×100.

**Figure 5:**
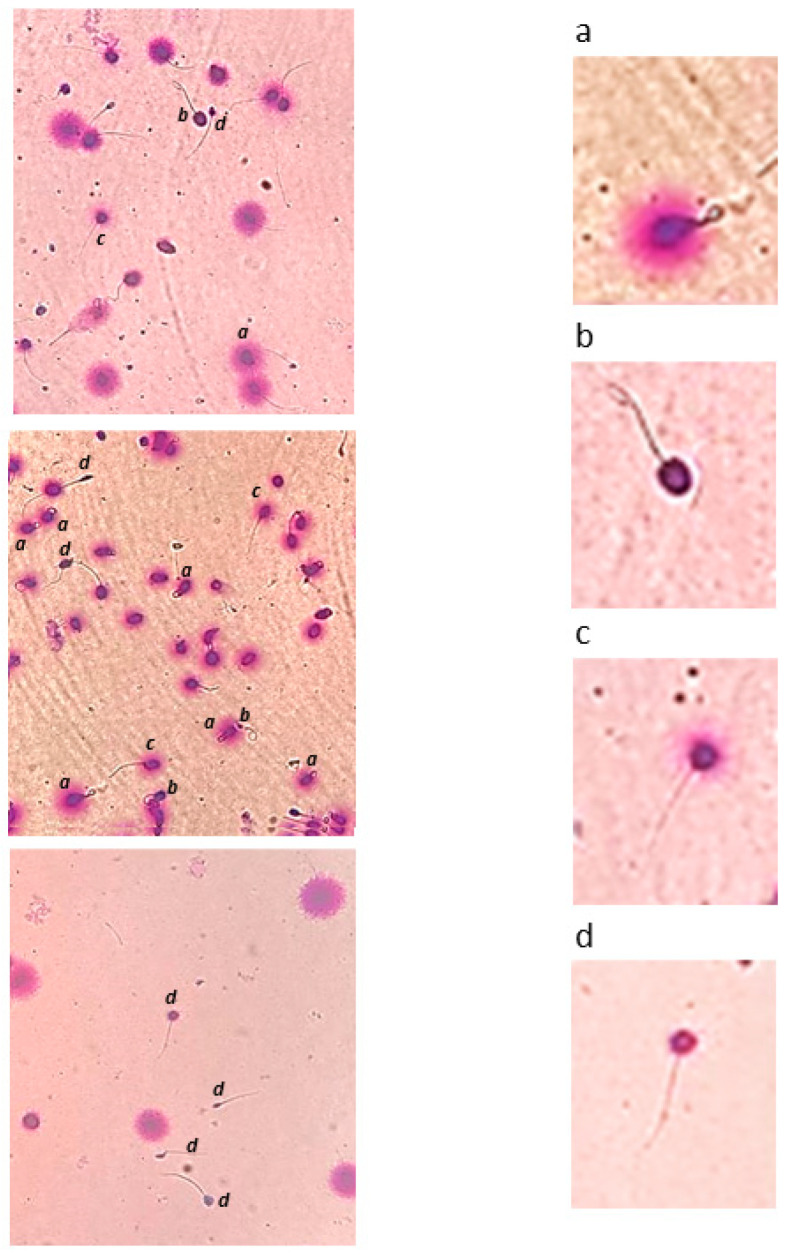
Left panels show images obtained under optical microscope of spermatozoa which were first incubated in hypo-osmotic medium (HOS) and then subjected to SCD test. Four patterns can be distinguished: (**a**) viable (curled tail) non-fragmented (SCD+); (**b**) viable (curled tail) fragmented (SCD−); (**c**) unviable (no curled tail) non-fragmented (SCD+); (**d**) unviable (no curled tail) fragmented (SCD−). An enlargement of each pattern is shown in the right panels.

In a subgroup of 52 samples, spermatozoa were incubated in a hypo-osmotic swelling (HOS) solution before performing the SCD in order to discriminate viable from unviable spermatozoa. Briefly, the samples were immersed for 1 h at 37 °C in hypo-osmotic medium [20] to induce swelling of the tail only in viable cells. Then, spermatozoa were processed as described above. This modified version of the SCD test will henceforth be referred as HOS/SCD. In this subgroup of samples, four sperm patterns were distinguished: (a) viable (curled tail) non-fragmented (SCD+) spermatozoa; (b) viable (curled tail) fragmented (SCD−) spermatozoa; (c) unviable (no curled tail) non-fragmented (SCD+) spermatozoa; and (d) unviable (no curled tail) fragmented (SCD−) spermatozoa. Examples of the four sperm patterns are shown in Figure 5.

### 4.4. Statistical Analysis

Statistical analysis was performed using the Statistical Package for the Social Sciences version 29.0.2.0 (SPSS, Chicago, IL, USA) for Windows. After verifying via the Kolmogorov–Smirnov test that the data were non-normally distributed, they were expressed as median values and interquartile ranges (IQR). To compare groups, the Mann–Whitney test was used. Correlations were assessed by Spearman’s correlation test. A *p*-value of 0.05 was considered significant.

## 5. Conclusions

In conclusion, our data indicate that SCD and TUNEL/PI are not aligned in determining sDF. Indeed, the sDF amount revealed by SCD is lower with respect to that detected by TUNEL/PI and, as a novel finding, is largely associated with unviable spermatozoa and only minimally with viable ones. It is necessary to make a careful evaluation of which is the most suitable test for determining sDF, especially for male partners of couples undergoing ART. Clearly, for a clinical use, it is important to use reliable tests that reveal the damage which better impacts reproductive outcomes for the evaluation of sDF. Although SCD is a simple technique to be used in ART laboratories in terms of feasibility, it appears to be less informative [11,27,28]. On the contrary, although the TUNEL assay is much more elaborate for clinical routines, it appears to be more reliable in integrating semen analysis into diagnostic evaluation of male infertility.

## Figures and Tables

**Table 1:** List of studies reporting the comparison of sDF values obtained by SCD test and TUNEL assay.

References	SCD/TUNEL Comparison
Ribas-Maynou et al. 2013 [21]	Similar sDF values
Zhang et al. 2010 [31]	SCD higher sDF values
Javed et al. 2019 [32]	SCD higher sDF values
Grèze et al. 2019 [33]	TUNEL higher sDF values
Chohan et al. 2006 [34]	Similar sDF values
Garcia-Peiro et al. 2011 [35]	Similar sDF values
Feijó and Esteves 2014 [36]	SCD higher sDF values
Present study	TUNEL higher sDF values

**Table 2:** Median [IQR] of age, sexual abstinence and basal semen characteristics of 71 male subjects included in this study.

Parameter	Median [IQR]	N
Age (years)	38.0 [34.0–42.0]	71
Sexual abstinence (days)	4.0 [3.0–4.0]	71
pH	7.8 [7.6–7.8]	71
Sperm rapid progressive motility (%)	28.0 [16.0–36.0]	71
Sperm slow progressive motility (%)	26.0 [20.0–33.0]	71
Sperm non-progressive motility (%)	6.0 [5.0–7.0]	71
Sperm total motility (%)	62.0 [52.0–69.0]	71
Sperm concentration (×10^6^/mL)	44.0 [20.0–74.0]	71
Sperm number (×10^6^/ejaculate)	145.0 [86.0–276.2]	71
Sperm viability (%)	77.0 [65.8–85.0]	71
Sperm normal morphology (%)	3.0 [2.0–5.0]	71

## Data Availability

The data presented in this study are available on request from the corresponding author.
